# Melanoma sentinel node biopsy and prediction models for relapse and overall survival

**DOI:** 10.1038/sj.bjc.6605849

**Published:** 2010-09-21

**Authors:** A Mitra, C Conway, C Walker, M Cook, B Powell, S Lobo, M Chan, M Kissin, G Layer, J Smallwood, C Ottensmeier, P Stanley, H Peach, H Chong, F Elliott, M M Iles, J Nsengimana, J H Barrett, D T Bishop, J A Newton-Bishop

**Affiliations:** 1 Section of Epidemiology and Biostatistics, Leeds Institute of Molecular Medicine, St James's University Hospital, Beckett Street, Leeds LS97TF, UK; 2 Royal Surrey County Hospitals NHS Trust, Surrey, UK; 3 St Georges's Hospital Melanoma Unit, Blackshaw Road, Tooting, London, UK; 4 Southampton University Hospitals NHS Trust, Tremona Road, Southampton, UK; 5 Hull and East Yorkshire Hospitals NHS Trust, Castle Hill Road, Hull, UK; 6 Leeds Teaching Hospitals Trust, Beckett Street, Leeds, UK

**Keywords:** melanoma, prognosis, sentinel node biopsy, formalin-fixed tissue, osteopontin

## Abstract

**Background:**

To optimise predictive models for sentinal node biopsy (SNB) positivity, relapse and survival, using clinico-pathological characteristics and osteopontin gene expression in primary melanomas.

**Methods:**

A comparison of the clinico-pathological characteristics of SNB positive and negative cases was carried out in 561 melanoma patients. In 199 patients, gene expression in formalin-fixed primary tumours was studied using Illumina's DASL assay. A cross validation approach was used to test prognostic predictive models and receiver operating characteristic curves were produced.

**Results:**

Independent predictors of SNB positivity were Breslow thickness, mitotic count and tumour site. Osteopontin expression best predicted SNB positivity (*P*=2.4 × 10^−7^), remaining significant in multivariable analysis. Osteopontin expression, combined with thickness, mitotic count and site, gave the best area under the curve (AUC) to predict SNB positivity (72.6%). Independent predictors of relapse-free survival were SNB status, thickness, site, ulceration and vessel invasion, whereas only SNB status and thickness predicted overall survival. Using clinico-pathological features (thickness, mitotic count, ulceration, vessel invasion, site, age and sex) gave a better AUC to predict relapse (71.0%) and survival (70.0%) than SNB status alone (57.0, 55.0%). In patients with gene expression data, the SNB status combined with the clinico-pathological features produced the best prediction of relapse (72.7%) and survival (69.0%), which was not increased further with osteopontin expression (72.7, 68.0%).

**Conclusion:**

Use of these models should be tested in other data sets in order to improve predictive and prognostic data for patients.

Several clinico-pathological characteristics of primary melanoma have been identified as independent prognostic factors for relapse and overall survival (OS), including age, sex, tumour site, Breslow thickness, ulceration, mitotic count, vessel invasion, regression and the presence of tumour-infiltrating lymphocytes (TILs) (Johnson *et al*, 1985; Clark *et al*, 1989; Cochran *et al*, 2000; Balch *et al*, 2001). Although as yet no OS benefit has been demonstrated from sentinal node biopsy (SNB) (Morton *et al*, 2006), the SNB status has been determined to be the single most important prognostic factor for melanoma (Gershenwald *et al*, 1999; Ferrone *et al*, 2002; Gutzmer *et al*, 2008) and is used in the American Joint Committee on Cancer (AJCC) staging system for cutaneous melanoma.

Breslow thickness is usually used to identify patients for SNB, but it has been shown to have poor sensitivity and specificity in predicting positivity (Sondak *et al*, 2004). Several groups have attempted to identify predictors of SNB positivity using clinical and histological characteristics of the primary melanoma, but so far only thickness has been consistently identified (Sondak *et al*, 2004), and it therefore remains the most commonly used criterion in selecting patients for SNB. If SNB is limited to melanoma patients with a tumour thickness ⩾1 mm, then micrometastases (SNB positivity) are detected in around 24% (Lens *et al*, 2002). The purpose of this study was to identify characteristics of primary melanoma, which better predict SNB positivity; to identify predictors of relapse-free survival (RFS) and OS and also to identify genetic prognostic biomarkers allowing further insight into the biological pathways important in melanoma progression.

We have used Illumina's DASL (cDNA-mediated annealing, selection, extension and ligation) assay (Illumina, Service XS, Leiden, Netherlands) to investigate prognostic biomarkers in formalin-fixed paraffin-embedded (FFPE) primary melanomas. This is a novel assay designed specifically to generate reproducible RNA profiles from FFPE tissue, in which the extracted RNA is often significantly degraded: partially degraded RNA has breaks throughout the RNA transcript making it difficult to generate high-quality full-length cDNA. The DASL assay uses random priming during cDNA synthesis, which means it does not depend on an intact poly-A tail, and in addition it only requires the probes to span about 50 bases and is therefore adapted to degraded RNA (Illumina, 2005a, 2005b). Gene expression studies in FFPE melanomas have been relatively few in number, and most studies have predominantly used immunohistochemical staining and polymerase chain reaction techniques, allowing only a few genes to be analysed at a time. More recently, Winnepenninckx *et al* (2006) identified a 254-gene signature predictive of survival in 83 cryopreserved primary tumours, and Kauffmann *et al* (2008) identified increased expression of DNA repair genes in metastatic melanoma tumours from 60 tumour samples. Two small previous studies using cryopreserved tumours also showed a correlation between osteopontin expression and progression in melanoma (Zhou *et al*, 2005; Jaeger *et al*, 2007). We have recently reported the successful use of the DASL assay in melanoma primaries (Conway *et al*, 2009).

## Materials and methods

Patients with melanomas of Breslow thickness ⩾0.75 mm having undergone SNB were recruited to a multicentre retrospective study. A cutoff of 0.75 mm rather than 1 mm was chosen, as we were aware that participating centres had historically carried out SNBs for <1 mm melanomas based on other histological criteria, such as vertical growth phase, Clark's level IV/V and regression. The study was approved both by the UK national ethics committees (MREC: multicentre research ethics committee and PIAG: patient information advisory group), which determined that patients still under follow-up at the cancer centre should be consented to use of their tissues. Five centres from the United Kingdom (St George's Hospital, London; Royal Surrey County Hospital, Guildford; Southampton General Hospital, Southampton; Castle Hill Hospital, Hull and St James's University Hospital, Leeds) identified all patients having had SNB in their departments from November 1994 until 2006. The first 400 patients to have a positive SNB were selected. As more patients undergoing SNB have a negative than a positive SNB result, a subset of SNB-negative patients were randomly selected, frequency matched by year of SNB and by the centre at which the SNB was performed. Patients were excluded if they had previous cancers other than non-melanoma skin cancer or cervical carcinoma *in situ* or if they had multiple melanoma primaries. Sample size calculations based on 250 patients in each group showed that the minimum detectable odds ratio (OR) for predicting SNB positivity would be 2.2, with a power of 80% and a significance level of 0.001 assuming a risk factor prevalence of 0.4 and based on 50 independent factors being analysed to produce a study-wide significance level of 0.05.

Clinico-pathological characteristics were extracted from clinical files: age, sex, site of primary tumour, clinical maximal diameter of tumour as measured macroscopically by the pathologist, Breslow thickness, Clark's level, histological subtype, mitotic count (mm^−2^), presence or absence of ulceration, regression, vessel invasion, perineural invasion, TILs and microsatellites in either the primary or wider excision. In a proportion of the cases, factors such as ulceration (21.6%), regression (19.6%), vessel invasion (30.3%), perineural invasion (59.9%) and microsatellites (57.3%) were not mentioned in histology reports and were assumed to be absent for purpose of analysis. A sensitivity analysis on the completed data set for these five factors showed quantitatively similar results. Follow-up data were similarly extracted from clinical files. The date of first relapse in any site (local, in-transit, regional or distal) was used to calculate RFS. Relapse-free survival and OS were calculated from the date of primary diagnosis to time point of the recurrence or death or last follow-up.

### Tissue sampling/gene expression methods

Two hundred patients with a positive SNB who were first to undergo the procedure were identified, and the stored FFPE primaries were sought. We then randomly selected 100 of the patients with a negative SNB, from groups matched by SNB year, centre and sex, and their primary tumour blocks were also traced. A tissue microarray needle was then used to sample the advancing edge of the tumour (containing the lowest admixture of inflammatory or stromal cells) horizontally producing a 0.8 mm core of tumour as described previously (Conway *et al*, 2009). RNA was extracted from these tumour cores using the protocol in the High Pure Paraffin RNA Kit (Roche Diagnostics Ltd, Burges Hill, UK) and supplied to a service provider for gene expression studies using the Illumina DASL Cancer Panel, which targets 502 cancer genes with unique probes in three different locations per gene (Illumina, 2005b). Use of this technique by our group and the quality control measures used are described elsewhere (Conway *et al*, 2009).

### Statistical methodology

Predictors of SNB positivity, RFS and OS were identified by univariable and multivariable analyses using STATA version 9 (StataCorp 2007, College Station, TX, USA). Breslow thickness was examined as a categorical variable using the same classification as the AJCC staging system (Balch *et al*, 2003). Mitotic count was classified as absent, low (1–6 mm^−2^) or high (>6 mm^−2^) (Elder and Murphy, 1991). Tumour sites were grouped into four, as arms, head and neck, legs or trunk. Age and clinical diameter of the primary melanoma were analysed as continuous variables. For associations with SNB positivity, continuous variables were analysed using *t*-tests and categorical variables using *χ*^2^ contingency table tests. Simple logistic regression was used to obtain ORs and 95% confidence intervals (CIs). The determinants of RFS and OS were identified using Cox's proportional hazards model to give hazard ratios (HR) and 95% CI. Multiple forward and backward stepwise logistic regression analyses were then performed to identify independent predictors of SNB positivity, RFS and OS. A significance level of *P*<0.05 was used.

The gene expression data were normalised within BeadStudio Gene Expression Module v3.4 (Illumina, San Diego, CA, USA) before exporting to STATA version 9 for statistical analyses. Fluorescence intensities from Cy3 and Cy5 dyes were averaged for each probe and the expression level of each gene was computed as an average of the intensities from three probes. Background correction and cubic B-spline smoothing methods (Workman *et al*, 2002) were used and sample scaling was applied to remove the variation between plates. Samples that failed to express over 250 genes were classified as failed and excluded from further analysis. Mean gene expression was calculated for the sample replicates. Expression of each gene was transformed to a log_2_ scale so that an increase of one unit corresponds to a doubling of expression levels; comparison between samples categorised by histological variables was carried out using two-sample *t*-tests and regression. Survival analysis was performed using a separate Cox's proportional hazards model for each gene. Using a Bonferroni correction for multiple testing, the significance level was set at 0.0001 for these univariable analyses.

A cross-validation approach implemented in R (version 2.8.0.) (Team, 2010) was used to estimate the predictive ability of the different prognostic models. Ten percent of the data were excluded, the model fitted to the remaining 90% and the predictive ability of this model on the excluded 10% was evaluated. This process was repeated 1000 times, excluding a random 10% each time, and the results were averaged.

## Results

A combined total of 2044 sentinel node biopsies were carried out in the five participating centres, and 439 patients had a positive result (21.5%). From initial lists provided by the five centres, previously recorded data on Breslow thickness was available in 1570 patients (70%). Overall, 320 patients (20%) had an SNB carried out for a melanoma <1 mm, and from this group, 24 patients (7.5%) had a positive SNB.

After identification of SNB positive and negative patients as described above, a total of 701 patients’ case notes were reviewed, of whom 608 patients met the inclusion criteria. Of these 608 patients, 2 refused participation in the study and 45 did not reply to the invitation to participate, leaving 561 patients’ data sets available for analysis. The majority of these patients (73%) were recruited from St George's Hospital, Melanoma Unit London, where SNB had been carried out for the longest period. In total, 218 FFPE primary tumours were collected. Of these, 199 blocks were selected for sampling. Reasons for not sampling a block included too little residual tumour after sectioning for clinical purposes or other research projects, tumour cells being mixed with large numbers of normal stromal or inflammatory cells, or because the wrong blocks were sent. Only two (0.9%) of the extracted samples were classified as failed samples. One of these failed samples was a technical replicate, which left 198 tumour samples for analysis. The mean age of the blocks was 6.26 years (range 2.23–15.16 years). Increasing age of block was associated with decreased gene detection (Spearman's correlation −0.24, *P*=0.0002) (Conway *et al*, 2009).

Table 1 shows the clinico-pathological characteristics of the patients in the total study group and in the tumour subset. Owing to the selection process, sentinel lymph nodes were positive for tumours in 286 of 561 patients (51.0%) in the total study group and in 131 of 198 patients whose FFPE tumours were sampled (66.2%) (the tumour subset). In our study group, there were 68 patients with a Breslow thickness between 0.75 and 1 mm (14 had a positive SNB, 20.6%). The median follow-up time for the total study group was 29.5 and 38.4 months for the tumour subset.

### Prediction of SNB status

#### Clinico-pathological predictors of SNB positivity

Table 2 shows the results of univariable unadjusted *χ*^2^ analysis and age/sex-adjusted logistic regression analysis of clinico-pathological factors associated with SNB positivity. In single variable analyses, the clinico-pathological factors associated with an increased risk of SNB positivity were increasing Breslow thickness (overall χ^2^, *P*=<0.0001), Clark's level (overall χ^2^, *P*=0.001), higher mitotic count (overall χ^2^, *P*<0.0001), presence of ulceration (*P*=<0.0001), presence of vessel invasion (*P*=0.02), presence of microsatellites (*P*=0.007), tumour site (overall χ^2^, *P*=0.04) and histological subtype (overall χ^2^, *P*=0.005). Patients with tumours situated on the leg or trunk showed a higher risk of SNB positivity than those with tumours on the arm (*P*=0.02, 0.007, respectively). Patients with nodular melanomas showed a higher risk of SNB positivity compared with patients with superficial spreading melanomas (*P*=0.02). Factors associated with a decreased risk of SNB positivity were the presence of more TILs (*P*=0.04) and the presence of regression (*P*=0.05). Factors not found to be significantly associated with SNB positivity were age, sex, perineural invasion and clinical diameter of the primary melanoma.

Clark's level and histological subtype were mentioned in histology reports less frequently than other variables, indicating higher amounts of missing data. These variables were therefore not included in the multivariable analyses. However, both Clark's level and histological subtype showed a strong correlation with Breslow thickness (Spearman's correlation 0.4, *P*<0.00001), and in the subset of individuals without missing data they were not independently significantly associated with SNB positivity once adjustment was made for Breslow thickness alone. The remaining significant prognostic factors adjusting for age and sex were entered into a stepwise logistic regression model, which revealed that the independent predictors of SNB positivity were thickness, mitotic count and tumour site (Table 3). The results were the same using either forwards or backwards selection.

### DASL gene expression data and prediction of SNB positivity

The gene whose expression was most predictive of SNB positivity in univariable analysis in this study was osteopontin (*SPP1*), (OR 2.7 for each doubling of expression levels, 95% CI (1.8–4.1), *P*=2.4 × 10^−7^). This was the only gene on the DASL cancer panel to be significantly associated with SNB positivity following Bonferroni correction. This association persisted in multivariable analysis adjusting for age, sex and tumour site (OR 3.1, 95% CI (2.0–4.8), *P*=<0.0001), and when adjusted additionally for thickness, mitotic count, presence of ulceration and vessel invasion (OR 2.3, 95% CI (1.4–3.8), *P*=0.001). The fold change of expression signal was 1.6 between negative and positive SNB patients in unadjusted analysis. Osteopontin was also the gene most associated with increasing tumour thickness (*P*=3.15 × 10^−11^, Spearman's correlation 0.42). It also showed significant association with increasing mitotic count (*P*=0.0009, Spearman's correlation 0.24).

### ROC analysis of models to predict SNB positivity

**Figure 1:**
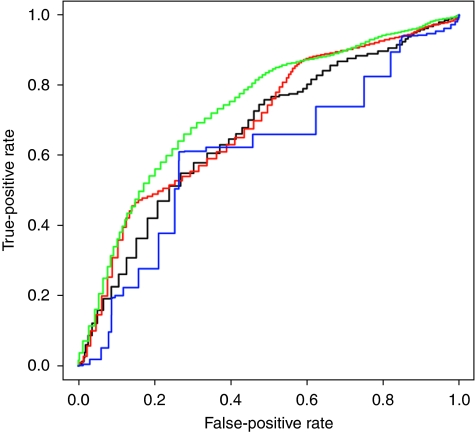
Receiver operating characteristic (ROC) analysis of predictors of SNB positivity for tumour subset group. Green: Breslow, mitotic rate, tumour site, age sex and SPP1 expression, AUC=72.6%. Red: Breslow, mitotic rate, tumour site, age and sex, AUC=68.6%. Black: SPP1 expression alone, AUC=65.7%. Blue: Breslow thickness alone, AUC=60.9%.

Using the total study group, we found that SNB positivity was best predicted by a model including thickness, mitotic count, tumour site, age and sex, giving an area under the curve (AUC) of 68.0%, compared with using a model using thickness alone, which gave an AUC of 58.0%. Figure 1 shows the receiver operating characteristic (ROC) curves for prediction of SNB positivity if osteopontin is included in the prognostic models using patients from the tumour subset. Osteopontin expression gave a better AUC (65.7%) than Breslow thickness alone (60.9%) in the tumour subset. Use of clinico-pathological features (thickness, mitotic count, site, age and sex) increased the AUC to 68.6%. However the best AUC of 72.6% was seen using a combination of osteopontin expression and the clinico-pathological variables together.

### Prognostic indicators

#### Clinico-pathological predictors of RFS and OS

Univariable clinical predictors of poor RFS included increasing age (HR 1.01 for each year, 95% CI (1.00–1.03), *P*=0.01), male sex (HR 1.6, 95% CI (1.1–2.4), *P*=0.007), increasing clinical diameter of the tumour (HR 1.03, 95% CI (1.01–1.05), *P*=<0.0001) and tumour site (*P*=0.007). Patients with tumours on the head and neck (HR 3.1, 95% CI (1.5–6.5), *P*=0.003) and tumours on the trunk (HR 2.0, 95% CI (1.1–3.5), *P*=0.02) were more likely to relapse than patients with tumours on the arm. Histological predictors of poorer RFS by univariable analysis included SNB positivity (HR 4.6, 95% CI (2.8–7.6), *P*=<0.0001), increasing Breslow thickness (*P*<0.0001), increasing mitotic count (*P*=<0.0001), increasing Clark's level (*P*=<0.0001), presence of ulceration (HR 2.7, 95% CI (1.9–3.9), *P*=<0.0001), presence of vessel invasion (HR 4.0, 95% CI (2.2–7.1), *P*=<0.0001) and the presence of microsatellites (HR 2.8, 95% CI (1.5–5.2), *P*=0.001). Improved RFS was seen with a brisk TILs reaction (HR 0.3, 95% CI (0.1–0.6), *P*=0.001).

Univariable clinical predictors of poor OS were male sex (HR 1.8, 95% CI (1.1–2.8), *P*=0.02), increasing tumour diameter macroscopically (HR 1.04, 95% CI (1.02–1.06), *P*=0.001) and tumour site (*P*=0.01). Patients with tumours on the trunk were significantly less likely to survive than patients with tumours on the arm (HR 2.8, 95% CI (1.3–5.9), *P*=0.009). Histological predictors of poorer OS by univariable analysis included SNB positivity (HR 5.9, 95% CI (2.8–12.3), *P*=<0.0001), increasing Breslow thickness (*P*<0.0001), increasing mitotic count (*P*=<0.0001), increasing Clark's level (*P*=<0.0001), presence of ulceration (HR 2.4, 95% CI (1.5–3.8), *P*=<0.0001), presence of vessel invasion (HR 2.9, 95% CI (1.3–6.4), *P*=0.01) and presence of microsatellites (HR 3.3 95% CI (1.6–7.0), *P*=0.001). Improved OS was seen with a brisk TILs reaction (HR 0.3, 95% CI (0.1–0.8), *P*=0.02).

Clinical diameter of the lesion was not included in the multivariable analysis of RFS and OS due to concerns about the accuracy of measurement. The clinical diameter of the lesion however showed strong correlation to Breslow thickness (Spearman's correlation 0.3, *P*<0.00001) and did remain significant after adjusting for Breslow thickness alone as a predictor of reduced RFS and OS. Clark's level was also once again not included in multivariable analysis of RFS and OS due to missing data, but was not significant as a predictor of reduced RFS or OS once adjustment for Breslow thickness was made. Table 3 shows multivariate stepwise logistic regression for RFS and OS showing that the SNB status was the most significant single predictor of both RFS and OS. Other independent predictors of RFS were thickness, ulceration and vessel invasion. Breslow thickness was also an independent predictor of OS.

### Gene expression data and prognosis

Increased osteopontin expression was also associated with reduced RFS (HR 1.6 for each doubling in expression levels, 95% CI (1.1–2.3), *P*=0.006) in unadjusted analyses, with a fold change of 1.32 between relapsers and non-relapsers. Osteopontin expression remained associated with RFS when corrected for age, sex and tumour site (*P*=0.006), but after adjusting additionally for SNB status did not reach statistical significance at the *P*=0.05 level (*P*=0.07). It did not remain significant in multivariable analysis with primary clinico-pathological characteristics (thickness, mitotic count, ulceration, vessel invasion) (Conway *et al*, 2009).

Increased osteopontin expression furthermore was associated with poorer OS (HR 1.6, 95% CI (1.1–2.5), *P*=0.02) in unadjusted analysis. The fold change between survivors and non-survivors was 1.3 (Conway *et al*, 2009). Osteopontin remained associated with OS after adjusting for age, sex and tumour site (*P*=0.02), but did not remain significant (*P*=0.07) after additionally adjusting for SNB status. It did not remain significant in multivariable analysis including primary clinico-pathological characteristics (thickness, mitotic count, ulceration, vessel invasion).

### ROC analysis of models to predict relapse and survival

**Figure 2:**
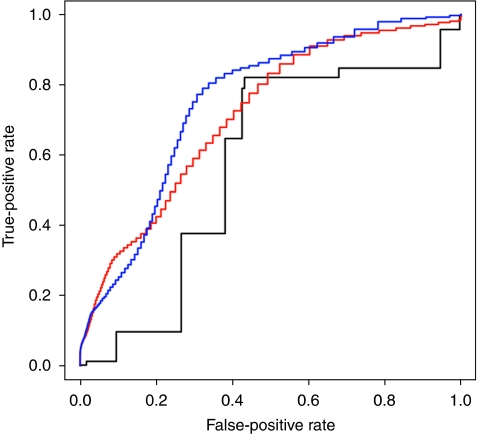
Receiver operating characteristic (ROC) analysis of predictors of relapse for the total study group. Blue: clinico-pathological features (Breslow, mitotic count, vessel invasion, ulceration, site, age, sex) and SNB status, AUC=76.0%. Red: clinico-pathological features alone, AUC=71.0%. Black: SNB status alone, AUC=57.0%.

Figure 2 shows the ROC curves of different prognostic models for prediction of relapse (yes/no) for the total study group. A model using SNB status alone gave an AUC of 57.0% for relapse and 55.0% for OS (not shown). The clinico-pathological model (thickness, mitotic count, ulceration, vessel invasion, site, age and sex) gave a better AUC of 71.0% for relapse and 70.0% for OS. Combining this model with SNB status produced a small increase to 76.0% for relapse and 74.0% for OS.

**Figure 3:**
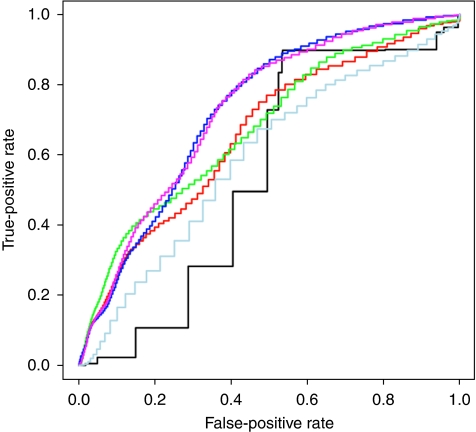
Receiver operating characteristic (ROC) analysis of predictors of relapse for tumour subset group. Purple: clinico-pathological features, SNB status, SPP1 expression, AUC=72.7%. Dark blue: clinico-pathological features, SNB status, AUC=72.7%. Green: clinico-pathological features and SPP1 expression, AUC=67.4%. Red: clinico-pathological features alone, AUC=66.2%. Light blue: SPP1 expression alone, AUC=59.0%. Black: SNB status alone, AUC=53.7%.

Figure 3 shows the ROC curves of different prognostic models to predict relapse in the smaller tumour subset group. The AUC for osteopontin expression combined with the clinico-pathological features predicted relapse (67.4%) and survival (67.5%) better than SNB status alone (53.7, 50.6%) or the clinico-pathological features alone (66.1 and 67.0%). SNB status combined with the clinico-pathological features produced a small increase to 72.7% for relapse and 69.0% for survival prediction, which was not increased further with osteopontin expression.

## Discussion

Several clinico-pathological characteristics have been inconsistently associated with SNB positivity in a number of studies, including Clark's level, ulceration, mitotic count, microsatellites, vessel invasion, TILs, tumour site and age (Mraz-Gernhard *et al*, 1998; Porter *et al*, 2000; Wagner *et al*, 2000; McMasters *et al*, 2001; Nguyen *et al*, 2001; Rousseau *et al*, 2003; Sondak *et al*, 2004; Kesmodel *et al*, 2005; Wong *et al*, 2005; Kruper *et al*, 2006; Paek *et al*, 2007; Taylor *et al*, 2007; Pinero *et al*, 2008; Sartore *et al*, 2008). Although some previous studies have showed that patients in younger age groups were more likely to have a positive sentinel node (McMasters *et al*, 2001; Rousseau *et al*, 2003; Sondak *et al*, 2004; Paek *et al*, 2007), we found no association between age and SNB status in our study when analysing age as either a continous or categorical variable (<50 or >50 years). Variation in identified predictors from previous studies may be due to small studies and different study populations (Sondak *et al*, 2004; Wong *et al*, 2005). In comparison with previous studies, our study has a large sample size of SNB- positive patients and in addition has also looked at gene expression in the primary melanoma to investigate prognostic biomarkers.

In our study, the only clinico-pathological independent predictors of SNB positivity were mitotic count, tumour site and thickness. Although Breslow thickness is usually used alone to identify patients eligible for SNB, we have shown that we can predict positivity more effectively if we combine clinico-pathological characteristics in a model, and therefore considering these factors together would likely be more helpful in counselling patients about SNB. Osteopontin gene expression was the only gene on the DASL cancer panel to be significantly associated with SNB positivity. By adding osteopontin expression to the clinico-pathological model, the best AUC of 72.6% was achieved.

Osteopontin is a secreted phosphorylated glycoprotein also known as secreted phosphoprotein-1 or SPP1. It was originally identified as a bone matrix protein and subsequently identified as a cytokine (Wang and Denhardt, 2008) and has been implicated in the prognosis of different cancers, such as breast, lung, prostate, colon, as well as melanoma (Rittling and Chambers, 2004; Rangaswami *et al*, 2006). Several mechanisms by which SPP1 could be involved in cancer progression have been suggested. The interaction between osteopontin and its receptors, such as integrins, *α*v*β*3 and CD44 (Wang and Denhardt, 2008), and epidermal growth factor receptor (Rangaswami *et al*, 2006) can lead to several signals including upregulation of metalloproteinase and stimulation of urokinase plasminogen activor. This forms a basis for metastatic potential by cell migration, cell adhesion and cell invasiveness (El-Tanani *et al*, 2006). Osteopontin is also an antiapoptotic factor and could promote cancer cell metastasis by preventing programmed cell death (Hsieh *et al*, 2006). *In vitro* studies have suggested a role for osteopontin in melanoma progression (Philip *et al*, 2001; Zhou *et al*, 2005). Very recently a large immunohistochemical study of 345 melanomas (256 with SNB status) also reported that increased osteopontin expression was an independent prognostic marker for melanoma being associated with SNB positivity, reduced RFS and OS (Rangel *et al*, 2008). The same authors have gone on to use osteopontin protein expression in a multimarker assay including two other markers not present on our DASL cancer panel (NCOA3, a member of the steroid receptor coactivator 1 family and RGS1, a GTPase-activating protein) and found the multimarker index to be the most significant factor in predicting RFS (Kashani-Sabet *et al*, 2009).

This study confirms that SNB is of strong prognostic value in melanoma patients with HRs for RFS and OS being similar to that seen in the third interim analysis of the multicentre-selective lymphadenectomy trial (Morton *et al*, 2006). SNB results are reported to provide a more accurate basis for formulating a prognosis than standard demographic and histopathological factors (Morton *et al*, 2006), and it is common in clinical practice to use SNB results alone to give prognostic information to melanoma patients. However, our study is the first to show that prognosis could be better predicted if clinicians used combined data from the pathology report of the primary tumour in a model rather than by using the SNB result. Combining SNB status with those clinico-pathological features, however, does produce a small further increase in prognostic predictive ability. These data therefore suggest that although SNB does improve prognostic estimates, the additional prognostic benefit from the operation is rather limited. Addition of osteopontin expression into a model did not further increase prognostic predictive ability once clinico-pathological features and SNB status had been considered in our data set.

Limitations of our multicentre study include involvement of many different pathologists, which can lead to variability in reporting. Cases were however generally reviewed by the melanoma multidisciplinary team pathology committee at each centre. The majority of cases furthermore (83%) originated from either St George's Hospital or The Royal Surrey hospital, where slides are reviewed by the same melanoma team involved in setting the EORTC guidance for pathological handling and assessment of sentinel nodes (Cook *et al*, 2003). However, as explained in the methodology some histological factors were assumed to be negative due to absence of reporting. Although a sensitivity analysis showed quantitavely similar results on the smaller completed data set, this is a limitation of the study. Patients were excluded from multivariable analysis due to incompleteness of pathological data. Owing to the retrospective nature of this study, the follow-up time was not standardised and varied considerably. Although the longest period of follow-up did reach 16.75 years, there were only three patients who had follow-up data over 10 years. We have used OS rather than melanoma-related survival in the analyses, as sufficient medical details, to be entirely sure of the cause of death, were not available in all non-survivors. However, 94% of deaths were known to be melanoma-related. With regard to gene-expression analysis, a main limitation of the study is the presence of a limited number of genes on the DASL Cancer Panel.

It was hoped that SNB would have therapeutic value for melanoma but there is no evidence currently that this is the case (Morton *et al*, 2006). The operation is well tolerated by the majority but is associated with morbidity (Nguyen *et al*, 2001), so that an estimate of the likelihood of positivity as reported here may help some patients to decide, whether to have the procedure or not. Our study adds further evidence that SNB positivity and outcome from melanoma is related to clinico-pathological features of the primary melanoma.

Although the results of SNB did add to prognostic estimates, our data suggest that it adds relatively little, if all the other clinico-pathological data are taken into account. The cost/benefit ratio from SNB therefore remains to be established. Although estimates of the predictive value of these models are given here, much larger pooled data analyses from many centres are necessary to develop robust predictive and prognostic models to allow use in clinical practice.

## Figures and Tables

**Table 1:** Clinico-pathological data in total study group and the subset of patients in whom primary tumours were sampled

**Variable**	**Total study group**	**FFPE tumour subset**
Total number of patients	561	198
Number of patients with positive SNB (%)	286 (51.0)	131 (66.2%)
Age at SNB years (mean and range)^a^	51.4 (7.0–88.7)	52.2 (14.8–88.1)
Sex – male (number and %)	275 (49.0)	103 (52.0)
		
*Site of tumour, number* (%)		
Arm	110 (19.6)	44 (22.2)
Head–neck	38 (6.8)	17 (8.6)
Leg	202 (36.0)	69 (34.9)
Trunk	211(37.6)	68 (34.3)
		
Breslow thickness, mm; median (range)	1.9 (0.75–24)	2.00 (0.8–24.0)
		
*Clark's level, number* (%)^b^		
I/II/III	160 (33.1)	52 (31.1)
IV	290 (60.1)	100 (59.9)
V	33 (6.8)	15 (9.0)
		
*Histological subtype* b		
Superficial spreading	193 (52.3)	50 (42.4)
Nodular	132 (35.8)	54 (45.8)
Other	44 (11.9)	14 (11.8)
		
*Mitotic count, number* (%)^b^		
<1	83 (18.8)	22 (12.2)
1–6	227 (51.3)	90 (50.0)
>6	132 (29.9)	68 (37.8)
		
Ulcerated tumours, number (%)^b^	142 (25.7)	58 (29.3)
Presence of vessel invasion, number (%)^b^	26 (4.7)	12 (6.1)
Presence of regression, number (%)^b^	73 (13.4)	18 (9.2)
Presence of perineural invasion, number (%)^b^	6 (1.1)	2 (1.0)
Presence of microsatellites, number (%)^b^	23 (4.2)	
		
*TILs, number* (%)^b^		
Absent	123 (25.0)	51 (27.9)
Non-brisk	264 (53.8)	90 (49.2)
Brisk	104 (21.2)	42 (22.9)
		
Median follow-up time months (range)	29.5 (0.03–201)	38.4 (0.03– 111.7)
		
Number of relapsers (%)	126 (23.0)	63 (32.8)
Number of deaths (%)	83 (14.9)	47 (24.0)

Abbreviations: FFPE=formalin-fixed paraffin-embedded; SNB=sentinel node biopsy; TIL=tumour-infiltrating lymphocyte.

a Only four patients under the age of 18 years in study.

b Clarks level available for 483 patients, histological subtype available for 369 patients, mitotic count available for 442 patients, ulceration available for 552 patients, vessel invasion available for 548 patients, regression available for 546 patients, perineural invasion available for 551 patients, microsatellites available for 550 patients, TILs data available for 491 patients. Relapse status available in 548 patients, survival status available in 559 patients.

**Table 2:** Clinico-pathological predictors of SNB positivity by univariable unadjusted *χ*^2^ analysis and age–sex-adjusted logistic regression analysis

**Factor**	**Factor groups**	**Overall *χ*^2^ *P*-value**	Adjusted for age and sex OR (95% CI)*	**Adjusted *P*-value**
Breslow (mm)	0.75–1	<0.0001	1.0	
	1.01–2		2.9 (1.5–5.5)	0.001
	2.01–4		7.5 (3.8–14.7)	<0.0001
	>4		9.9 (4.5–21.6)	<0.0001
Clark's level	I/II/III	0.001	1.0	
	IV		1.5 (1.0–2.2)	0.04
	V		5.7 (2.3–14.1)	<0.0001
Mitoses (mm^2^)	<1	<0.0001	1.0	
	1–6		3.5 (2.0–6.0)	<0.0001
	>6		6.7 (3.6–12.4)	<0.0001
Ulceration	Absent	<0.0001	1.0	
	Present		2.2 (1.5–3.3)	<0.0001
Vessel invasion	Absent	0.02	1.0	
	Present		2.7 (1.1–6.6)	0.03
Microsatellites	Absent	0.007	1.0	
	Present		3.5 (1.3–9.5)	0.02
Tumour site	Arm	0.04	1.0	
	Head/neck		1.5 (0.7–3.2)	0.3
	Leg		1.9 (1.2–3.0)	0.01
	Trunk		1.8 (1.1–2.9)	0.02
Tumour subtype	SSM	0.005	1.0	
	Nodular		2.1 (1.3–3.3)	0.002
	Other		1.0 (0.5–1.9)	1.0
TILs	Absent	0.07	1.0	
	Non-brisk		0.9 (0.6–1.4)	0.7
	Brisk		0.6 (0.3–1.0)	0.05
Regression	Absent	0.06	1.0	
	Present		0.6 (0.4–1.0)	0.05

Abbreviations: CI=confidence interval; OR=odds ratio; SNB=sentinel node biopsy; SSM=superficial spreading melanomas; TIL=tumour-infiltrating lymphocyte.

**Table 3:** Multivariable stepwise logistic regression analyses of clinico-factors associated with SNB positivity, RFS and OS

**Analysis**	**Factor**	**Factor groups**	**OR (95% CI) for SNB positivity HR (95% CI) for RFS/OS**	***P*-value**
SNB positivity^a^	Breslow thickness (mm)	0.75–1	1.0	
		1.01–2	1.6 (0.7–3.5)	0.3
		2.01–4	2.9 (1.3–6.9)	0.01
		>4	3.4 (1.3–9.3)	0.02
	Mitotic count (mm^−2^)	0	1.0	
		0.1–6	2.7 (1.5–5.1)	0.002
		>6	4.2 (2.0–8.7)	<0.0001
	Tumour site	Arm	1.0	
		Head/neck	1.9 (0.7–4.7)	0.2
		Leg	2.4 (1.3–4.3)	0.005
		Trunk	2.6 (1.4–4.8)	0.002
RFS^b^	SNB status	Negative	1.0	
		Positive	2.5 (1.5–4.3)	0.001
	Breslow thickness (mm)	0.75–1	1.0	
		1.01–2	2.2 (0.6–7.2)	0.2
		2.01–4	2.2 (0.6–7.4)	0.2
		>4	5.3 (1.5–18.2)	0.009
	Ulceration	Absent	1.0	
		Present	1.6 (1.0–2.5)	0.03
	Vessel invasion	Absent	1.0	
		Present	3.0 (1.4–6.3)	0.003
	Tumour site	Arms	1.0	
		Legs	5.0 (2.0–12.5)	0.001
		Head and neck	1.5 (0.7–3.3)	0.3
		Trunk	2.5 (1.2–5.2)	0.02
OS^c^	SNB status	Negative	1.0	
		Positive	4.6 (1.9–10.8)	0.001
	Breslow thickness (mm)	0.75–1	1.0	
		1.01–2	1.5 (0.3–6.5)	0.6
		2.01–4	1.7 (0.4–7.4)	0.5
		>4	4.8 (1.1–21.1)	0.04

Abbreviations: CI=confidence interval; HR=hazards ratio; OR=odds ratio; OS=overall survival; RFS=relapse-free survival; SNB=sentinel node biopsy; TIL=tumour-infiltrating lymphocyte.

a Included 408 patients in analysis,

b Included 412 patients in analysis,

c Included 409 patients in analysis.
